# Multilevel determinants of knowledge on TB transmission among women of reproductive age

**DOI:** 10.5588/ijtldopen.26.0137

**Published:** 2026-07-13

**Authors:** M.F. Lozada-Urbano, A. Toledo Cornejo, C. Copaja-Corzo, J.R. Lucas, F. Huamán, F. Vasquez Vasquez, V. De La Cruz, L. Paucar Suasnabar, M. Merejildo Vera, J.C. Benites Azabache, J.D. Rojas

**Affiliations:** 1 Programa Académico de Nutrición y Dietética, Universidad Privada Norbert Wiener, Lima, Peru;; 2 Escuela de Enfermería, Universidad Científica del Sur, Lima, Peru;; 3 Unidad de Investigación para la Generación y Síntesis de Evidencias en Salud, Universidad San Ignacio de Loyola, Lima, Peru;; 4 School of Veterinary Medicine, Universidad Nacional Mayor de San Marcos, San Borja, Lima, Peru;; 5 Facultad de Ingeniería Económica, Estadística y Ciencias Sociales, Universidad Nacional de Ingeniería, Rimac, Peru;; 6 Centro de Investigación Interdisciplinaria en Sexualidad, Sida y Sociedad, Universidad Peruana Cayetano Heredia, Lima, Peru;; 7 Programa Académico de Tecnología Médica en Laboratorio Clínico y Anatomía Patológica, Facultad de Ciencias de la Salud, Universidad Privada Norbert Wiener, Lima, Peru.

**Keywords:** tuberculosis, Peru, health surveys, spatial analysis, socio-economic factors, health care disparities, cross-sectional studies

## Abstract

**BACKGROUND:**

In Peru, TB remains a leading public health concern, yet knowledge about its transmission among women of reproductive age is poorly characterised. We sought to determine multilevel factors associated with comprehensive TB knowledge in this population.

**METHODS:**

We analysed data from 29,889 women aged 15–49 years across 25 departments using Peru’s 2024 Demographic and Health Survey. Comprehensive TB knowledge was defined as correct identification of airborne transmission and rejection of at least three of five common myths. Three-level logistic regression, spatial autocorrelation, and concentration index analyses were used to examine individual, household, and contextual determinants.

**RESULTS:**

Weighted prevalence of comprehensive TB knowledge was 27.5% (95% confidence interval: 26.3–28.6). Education exhibited a dose–response gradient (odds ratio 4.7 for higher vs. no education; 95% confidence interval: 3.30–6.57). The intraclass correlation coefficient was 22.2%, indicating substantial contextual variation. Spatial analysis showed significant clustering (Moran’s I = 0.077, *P* < 0.001), and the concentration index of 0.115 confirmed pro-rich inequality in knowledge distribution.

**CONCLUSION:**

Approximately one in four Peruvian women possess comprehensive TB knowledge, with pronounced socio-economic and geographic inequities. These findings support targeted educational interventions for women with limited schooling in economically disadvantaged communities.

TB remains one of the world’s deadliest infectious diseases, with an estimated 10.7 million incident cases globally in 2024.^1^ In the Americas, the incidence rate increased for the fourth consecutive year in 2024.^2^ Peru remains classified as endemic (100–299 per 100,000) on the WHO high-TB-burden country list^3^; notwithstanding substantial investment in TB control, progress towards End TB Strategy targets has been hindered by complex social and structural determinants.^3^ Knowledge about TB transmission is directly linked to care-seeking behaviour and diagnostic delay.^4,5^ Systematic reviews have documented inadequate TB knowledge in low- and middle-income countries, with educational attainment, media exposure, rural residence, and socio-economic status consistently associated with awareness levels.^5–7^ Additionally, population-level TB knowledge influences the interval between symptom onset and diagnosis, a period during which transmission continues.^8,9^

Women of reproductive age merit particular attention: An estimated 3.7 million women developed TB in 2024, representing 35% of the global total.^1^ Gender-related barriers, including caregiving responsibilities, limited autonomy, and restricted health care access, contribute to delayed diagnosis.^1,10^ Furthermore, TB-associated stigma compounds these disadvantages, as women may conceal symptoms or avoid seeking care due to social consequences.^5,11^ In Peru, gender inequalities intersect with poverty, rurality, and indigenous identity, amplifying barriers to TB information and services.^10^ Individual-level determinants of TB knowledge operate within broader household, community, and regional contexts; geographic location and community-level poverty independently influence knowledge even after controlling for individual characteristics.^8,9^ These hierarchical relationships necessitate multilevel analytical approaches.

We sought to determine the multilevel determinants of TB transmission knowledge among women of reproductive age in Peru using 2024 Demographic and Health Survey data (n = 29,889), quantifying individual and contextual contributions and identifying priority subgroups for targeted interventions.

## METHODS

We conducted a cross-sectional study using secondary data from the 2024 Peruvian Demographic and Health Survey (Encuesta Demográfica y de Salud Familiar, ENDES), administered by the Instituto Nacional de Estadística e Informática (INEI). ENDES is a nationally representative household survey employing a stratified two-stage cluster sampling design, with primary sampling units (clusters) selected with probability proportional to size within strata defined by department and urban/rural area, followed by systematic household selection. The ENDES primarily monitors demographic dynamics, maternal and child health indicators, and communicable and non-communicable disease prevalence within Peru’s national results-based budgeting framework. This study is reported in accordance with the Strengthening the Reporting of Observational Studies in Epidemiology (STROBE) guidelines (Supplementary Data Table S1).

### Study population

All women aged 15–49 years with complete interviews and valid responses to the TB knowledge module were included. After excluding records with missing data on the primary outcome, the sample comprised 29,889 women nested within 3,074 clusters across 25 departments.

### Outcome variable

Comprehensive TB knowledge was defined as a composite binary indicator requiring: 1) correct identification of airborne transmission; 2) rejection of at least three of five TB-related myths (transmission through sharing utensils, physical contact, sharing food, sexual contact, or mosquito bites); 3) knowledge that TB is curable; and 4) a non-stigmatising attitude, defined as not keeping a family member’s TB diagnosis secret. Two alternative definitions were used in sensitivity analyses: a strict definition requiring rejection of all five myths and a relaxed definition requiring rejection of at least two.

### Covariates

Individual-level covariates included age group (15–24, 25–34, 35–44, 45–49 years), education level (none, primary, secondary, higher), media exposure (none, 1 source, 2–3 sources), wealth index quintile, improved water source, and improved sanitation. Contextual covariates included area of residence (urban/rural), altitude (per 1,000 m), natural region (Coast, Highlands, Jungle), and department-level TB incidence rate per 100,000 population (Ministerio de Salud, 2021; Supplementary Data Figure S1). A health care access barriers score (range 0–9) was constructed from nine binary items addressing perceived barriers to seeking care.

### Statistical analysis

Descriptive analysis: survey-weighted prevalence estimates with 95% confidence intervals (CIs) were computed accounting for the complex sampling design, stratified by socio-demographic characteristics, department, and natural region.

Multilevel modelling: given the hierarchical data structure, three-level logistic regression models were fitted with individuals (level 1) nested within clusters (level 2) nested within departments (level 3). Survey weights were incorporated using Carle’s scaling method,^12^ which rescales design weights within each cluster so that they sum to the cluster-specific sample size, thereby preserving relative weight ratios while avoiding inflation of effective sample sizes in maximum likelihood estimation. Six models were fitted sequentially: a null model (M0) to estimate the intraclass correlation coefficient (ICC), followed by models progressively adding individual (M1), household (M2), cluster (M3), and department-level predictors including TB incidence (M4). An additional model excluding TB incidence (M5) enabled comparison of model fit. Models were evaluated using Akaike Information Criterion (AIC), Bayesian Information Criterion, and Nakagawa’s marginal and conditional R^2^ values. Multicollinearity was assessed using variance inflation factors.

Spatial analysis: global spatial autocorrelation of cluster-level TB knowledge prevalence was assessed using Moran’s I statistic with a k-nearest neighbours spatial weights matrix (k = 8). Local Indicators of Spatial Association (LISA)^13^ identified statistically significant spatial clusters, including hotspots (High–High) and coldspots (Low–Low), as well as spatial outliers. Benjamini–Hochberg false discovery rate (FDR) correction was applied for multiple comparisons.

Health inequality analysis: socio-economic inequality in TB knowledge was quantified using the Concentration Index,^14^ computed via weighted covariance with survey-weighted fractional ranks. A positive index indicates pro-rich inequality. A 95% CI was obtained through cluster-based bootstrap resampling within strata (1,000 replications).

Sensitivity and exploratory analyses: sensitivity analyses included: 1) refitting the full model with strict and relaxed outcome definitions; 2) comparing weighted versus unweighted models; and 3) comparing three-level versus two-level specifications. Exploratory analyses included causal mediation analysis, interaction analysis between education and barriers, Latent Class Analysis (LCA), and Random Forest classification.

All analyses were performed in R (version 4.5.2) using lme4, survey, spdep, and sf packages.

### Ethical statement

The ENDES is a publicly available, de-identified dataset; therefore, institutional review board approval was not required for this secondary analysis.

## RESULTS

The analytical sample comprised 29,889 women nested within 3,074 clusters across 25 departments. Table 1 presents sample characteristics stratified by TB knowledge status; 47.1% had completed secondary education, and 82.8% resided in urban areas. Complete variable definitions are provided in Supplementary Data Table S2.

**Table 1.** Socio-demographic characteristics of the study sample.

Characteristic	n (%)^A^	95% CI
Age group		
15–24	8,344 (28.7)	27.7–29.7
25–34	10,476 (28.6)	27.5–29.7
35–44	8,458 (29.1)	28.0–30.2
45–49	2,611 (13.7)	12.8–14.6
Education level		
No education	336 (0.9)	0.8–1.1
Primary	4,492 (12.2)	11.6–12.9
Secondary	14,888 (47.1)	45.9–48.4
Higher	10,173 (39.7)	38.5–41.0
Media exposure		
No media	1,320 (4.0)	3.6–4.6
1 source	4,503 (13.8)	12.9–14.8
2–3 sources	24,066 (82.1)	81.1–83.1
Wealth index		
Poorest	8,566 (17.3)	16.6–18.1
Poorer	7,696 (21.3)	20.3–22.4
Middle	6,072 (21.5)	20.3–22.7
Richer	2,968 (19.6)	18.2–21.0
Richest	4,587 (20.3)	19.1–21.5
Improved water source	26,745 (91.6)	90.7–92.4
Improved sanitation	23,313 (85.7)	84.8–86.6
Area of residence		
Urban	20,883 (82.8)	82.2–83.4
Rural	9,006 (17.2)	16.6–17.8
Natural region		
Coast	12,745 (62.3)	61.1–63.4
Highlands	11,266 (28.5)	27.6–29.4
Jungle	5,878 (9.3)	8.9–9.7

Percentages may not sum to 100 due to rounding.

Data source: Peru Demographic and Health Survey (ENDES) 2024.

CI = confidence interval.

A Unweighted n; survey-weighted percentages.

**Figure 1.**
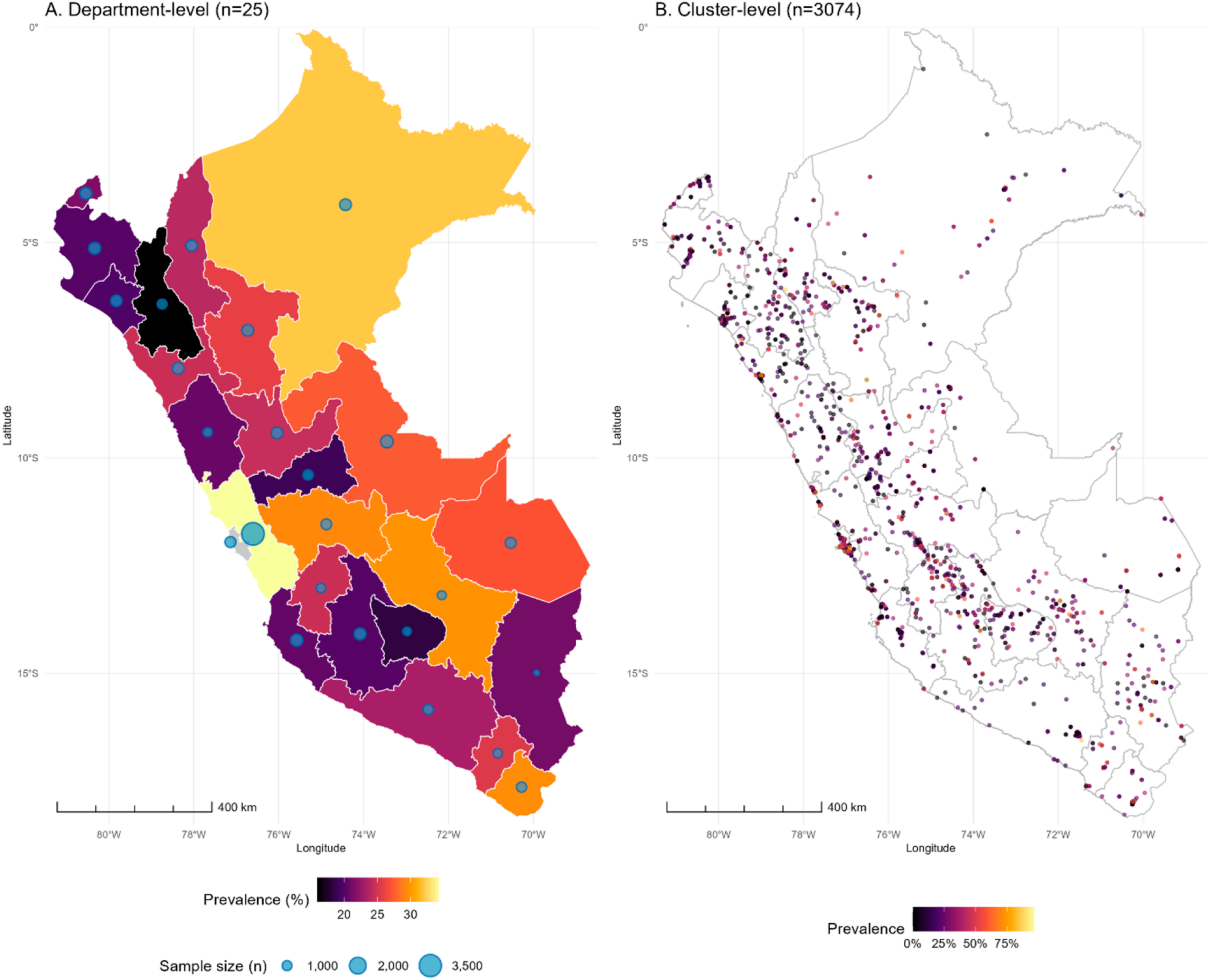
Geographic distribution of comprehensive TB knowledge prevalence among women of reproductive age, Peru Encuesta Demográfica y de Salud Familiar (ENDES) 2024. **A:** Survey-weighted prevalence (%) by department (n = 25). Departments are shaded from light (lowest: ∼16.0% in Cajamarca) to dark (highest: ∼34.2% in Lima). Circles indicate department-level sample size. **B:** Cluster-level prevalence based on 3,074 survey clusters, illustrating substantial within-department heterogeneity. Comprehensive knowledge was defined as correct identification of airborne transmission, rejection of ≥3/5 myths, knowledge of curability, and a non-stigmatising attitude.

The weighted prevalence of comprehensive TB knowledge was 27.5% (95% CI: 26.3–28.6). Individual components showed higher prevalences: 64.8% correctly identified airborne transmission, 67.6% knew TB is curable, and 58.6% expressed non-stigmatising attitudes (Table 2). Regional variation was observed, with the Coast showing the highest prevalence (29.4%, 95% CI: 27.7–31.2), followed by the Jungle (27.9%, 95% CI: 26.0–29.9) and Highlands (23.0%, 95% CI: 21.7–24.3). Department-level prevalences ranged from 16.0% in Cajamarca to 34.2% in Lima. Figure 1 illustrates substantial within-department heterogeneity.

**Table 2.** Prevalence of TB knowledge and its components by region (%), Peru ENDES 2024.

Component	Overall (95% CI)	Coast (95% CI)	Highlands (95% CI)	Jungle (95% CI)
Correct transmission (airborne)	64.8 (63.6–66.0)	66.6 (64.9–68.3)	63.0 (61.5–64.5)	58.2 (56.2–60.2)
Rejects myth: sharing utensils	64.5 (63.3–65.6)	64.2 (62.5–65.9)	67.8 (66.2–69.3)	55.9 (53.6–58.3)
Rejects myth: physical contact	93.4 (92.7–94.0)	93.2 (92.3–94.2)	92.9 (92.1–93.7)	95.7 (94.7–96.6)
Rejects myth: sharing food	95.8 (95.4-–96.3)	96.6 (96.0–97.3)	94.7 (94.0–95.5)	93.8 (92.8–94.8)
Rejects myth: sexual contact	92.3 (91.7–92.9)	91.9 (91.0–92.9)	93.2 (92.4–94.0)	92.1 (90.8–93.4)
Rejects myth: mosquitoes	99.9 (99.8–100.0)	99.8 (99.7–100.0)	99.9 (99.9–100.0)	100.0 (99.9–100.0)
Myth score ≥3/5	64.5 (63.3–65.7)	66.4 (64.7–68.2)	62.5 (61.0–64.0)	57.8 (55.8–59.8)
TB is curable	67.6 (66.4–68.7)	68.0 (66.4–69.6)	64.1 (62.6–65.7)	74.8 (72.9–76.8)
Non-stigmatising attitude	58.6 (57.4–59.8)	59.5 (57.7–61.2)	55.2 (53.6–56.7)	63.2 (61.1–65.3)
Comprehensive knowledge (main)	27.5 (26.3–28.6)	29.4 (27.7–31.2)	23.0 (21.7–24.3)	27.9 (26.0–29.9)
Comprehensive knowledge (strict)	14.2 (13.3–15.1)	15.8 (14.5–17.1)	11.7 (10.7–12.7)	11.1 (9.8–12.5)
Comprehensive knowledge (relaxed)	27.6 (26.4–28.7)	29.5 (27.8–31.2)	23.1 (21.8–24.4)	28.2 (26.3–30.2)

All estimates are survey-weighted accounting for the complex sampling design (stratification, clustering, and sampling weights).

Data source: Peru Demographic and Health Survey (ENDES) 2024.

CI = confidence interval.

The null multilevel model revealed an ICC of 22.2%, indicating that over one fifth of the variance in TB knowledge was attributable to contextual levels. The full model (M4) explained 7.8% of the variance through fixed effects (marginal R^2^ = 0.078) and 26.7% including random effects (conditional R^2^ = 0.267). Model diagnostics confirmed adequate fit and absence of multicollinearity (Supplementary Data Tables S3–S5). Normality of department-level random effects was supported by the Shapiro–Wilk test (W = 0.965, *P* = 0.526).

**Figure 2.**
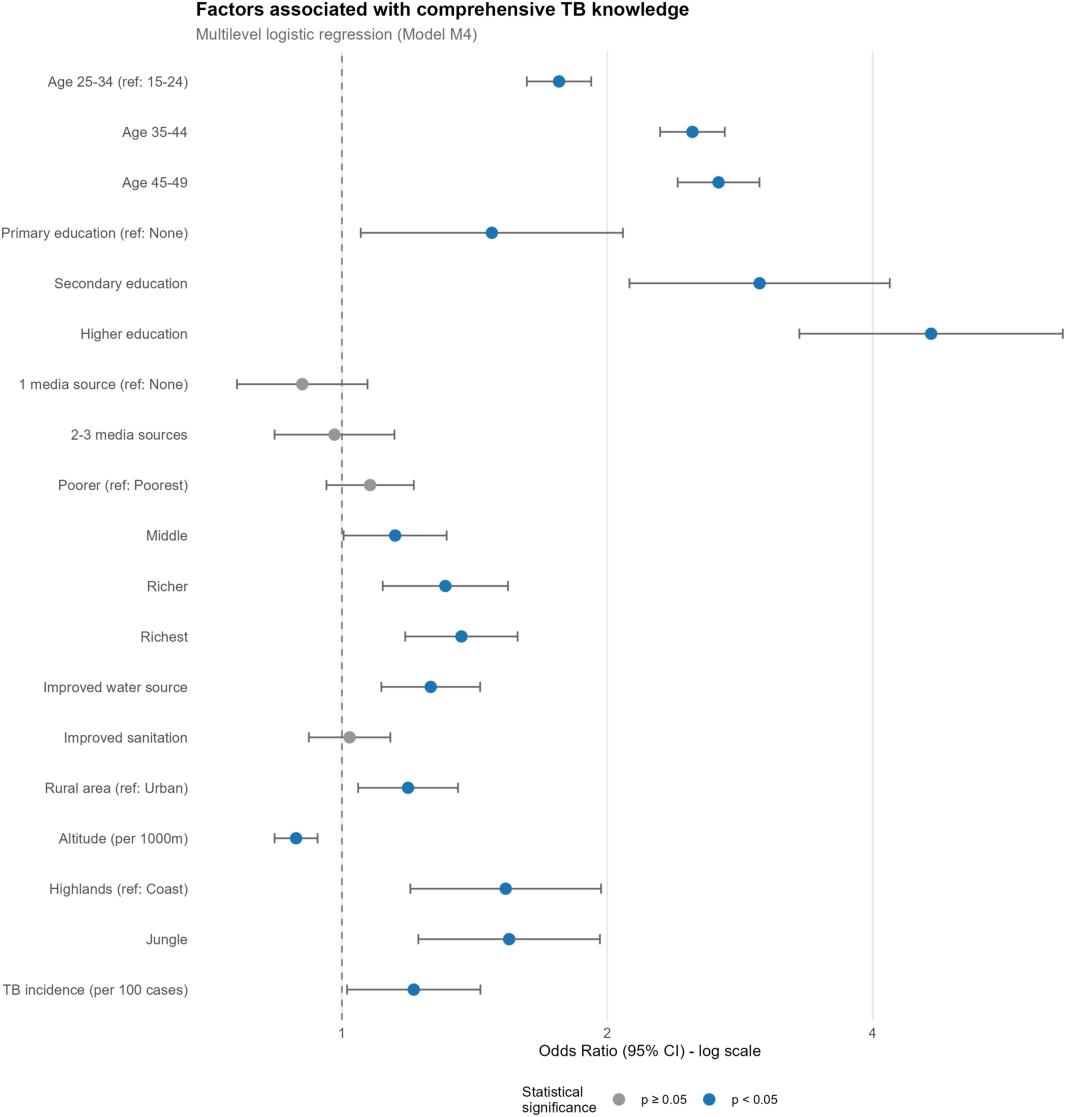
Forest plot of adjusted ORs from the full multilevel logistic regression model for factors associated with comprehensive TB knowledge among women of reproductive age, Peru Encuesta Demográfica y de Salud Familiar (ENDES) 2024. The model comprises three levels: individuals (n = 29,889) nested within clusters (n = 3,074) nested within departments (n = 25). Horizontal bars represent 95% CIs. The dashed vertical line indicates OR = 1.0 (no association). Blue points indicate statistically significant associations (*P* < 0.05); grey points indicate non-significant associations. Reference categories: age 15–24 years, no education, no media sources, poorest wealth quintile, urban area, and Coast region. Survey weights were incorporated using Carle’s scaling method. OR = odds ratio; CI = confidence interval.

Figure 2 displays adjusted odds ratios (ORs) from model M4. Education demonstrated the strongest association, a dose–response gradient: compared to women with no formal education, those with primary education had 1.5 times higher odds (95% CI: 1.05–2.08), those with secondary education 3.0 times higher (95% CI: 2.12–4.18), and higher education 4.7 times higher (95% CI: 3.30–6.57). Women aged 45–49 had 2.7 times higher odds than those aged 15–24 (95% CI: 2.40–2.98). The richest wealth quintile showed 1.4 times higher odds than the poorest (95% CI: 1.18–1.58; Supplementary Data Figure S2). Department-level TB incidence was positively associated with knowledge (OR = 1.21 per 100 cases/100,000; 95% CI: 1.01–1.44; *P* = 0.035), while altitude was inversely associated (OR = 0.89 per 1,000 m; 95% CI: 0.84–0.94; *P* < 0.001). Full model coefficients across all specifications (M0–M5) are detailed in Supplementary Data Table S6.

Spatial analysis revealed significant positive autocorrelation (Moran’s I = 0.077, *P* < 0.001), indicating geographic clustering of TB knowledge (Supplementary Data Figure S3). LISA identified 11 statistically significant hotspots of high TB knowledge (0.4%) and 7 low-high spatial outliers after FDR correction (Supplementary Data Table S7); notably, no coldspots (low-low clusters) were detected, indicating that low TB knowledge is spatially dispersed rather than geographically concentrated. Sensitivity analyses varying k from 4 to 20 confirmed robustness (Supplementary Data Table S8).

Sensitivity analyses using alternative outcome definitions yielded consistent results (Supplementary Data Tables S9 and S10): strict definition produced a prevalence of 14.2% and the relaxed definition 27.6%, with associations remaining stable across definitions. Weighted and unweighted models showed consistent results (Supplementary Data Tables S6 and S11), and M4 (including TB incidence) provided superior fit compared to M5 (AIC: 26,675.0 vs. 26,676.9; Supplementary Data Table S4). Random Forest variable importance ranking identified altitude, age, and barriers as top predictors (Supplementary Data Figure S4), and LCA identified three population subgroups with heterogeneous knowledge-barrier profiles (Supplementary Data Tables S12 and S13; Figure S5). Additional exploratory analyses are also available (Supplementary Data Tables S14–S20; Figures S6–S10) and model diagnostics (Supplementary Data Table S5; Figures S11 and S12).

## DISCUSSION

We sought to determine the multilevel determinants of comprehensive TB knowledge among women of reproductive age in Peru. In this dataset, only 27.5% (95% CI: 26.3–28.6) of women possessed comprehensive knowledge, a figure that places Peru in the lower-middle range internationally. Comparable Demographic and Health Survey analyses report 26.9% in Timor-Leste,^15^ 30.1% in Bangladesh,^16^ and 29.3% in Nigeria,^17^ while Ghana (43.2%)^8^ and Myanmar (44.6%)^9^ show considerably higher levels. India’s longitudinal data demonstrate that comprehensive knowledge rose from 30% in 2005–2006 to 70.4% in 2019–2021,^18,19^ suggesting that sustained interventions can substantially improve awareness. The 5–10 percentage point gender gap documented internationally^8,15,20^ further supports the need for gender-responsive strategies, particularly given the association between limited TB knowledge and delayed health care–seeking.

Community-based evidence has shown that delayed health care–seeking among individuals with TB-suggestive symptoms is associated with limited education, lower income, marital status, and poor TB knowledge.^5^

Education emerged as the strongest determinant in our analysis, exhibiting a dose–response gradient consistent with global evidence.^16,17,21^ Compared to women without formal education, odds increased from 1.5 for primary to 4.7 for higher education; these magnitudes align with estimates reported in Ethiopia (OR 3.28–7.42)^21^ and India,^19^ confirming education as a central pathway through which structural inequality shapes health literacy. Inverse association between altitude and knowledge (OR 0.89 per 1,000 m, 95% CI: 0.84–0.94) likely reflects not only geographic barriers to health services,^4,22^ but also linguistic barriers, as indigenous language predominance (Quechua and Aymara) strongly correlates with altitude in Peru.^23^ If health education materials are not delivered in contextually appropriate languages, knowledge may not reach these populations regardless of formal educational attainment, a pattern documented across indigenous communities in Latin America.^24^ Age showed an independent positive association, with women aged 45–49 exhibiting 2.7 times higher odds than those aged 15–24 (95% CI: 2.40–2.98), potentially reflecting cumulative exposure to health messaging.

**Figure 3.**
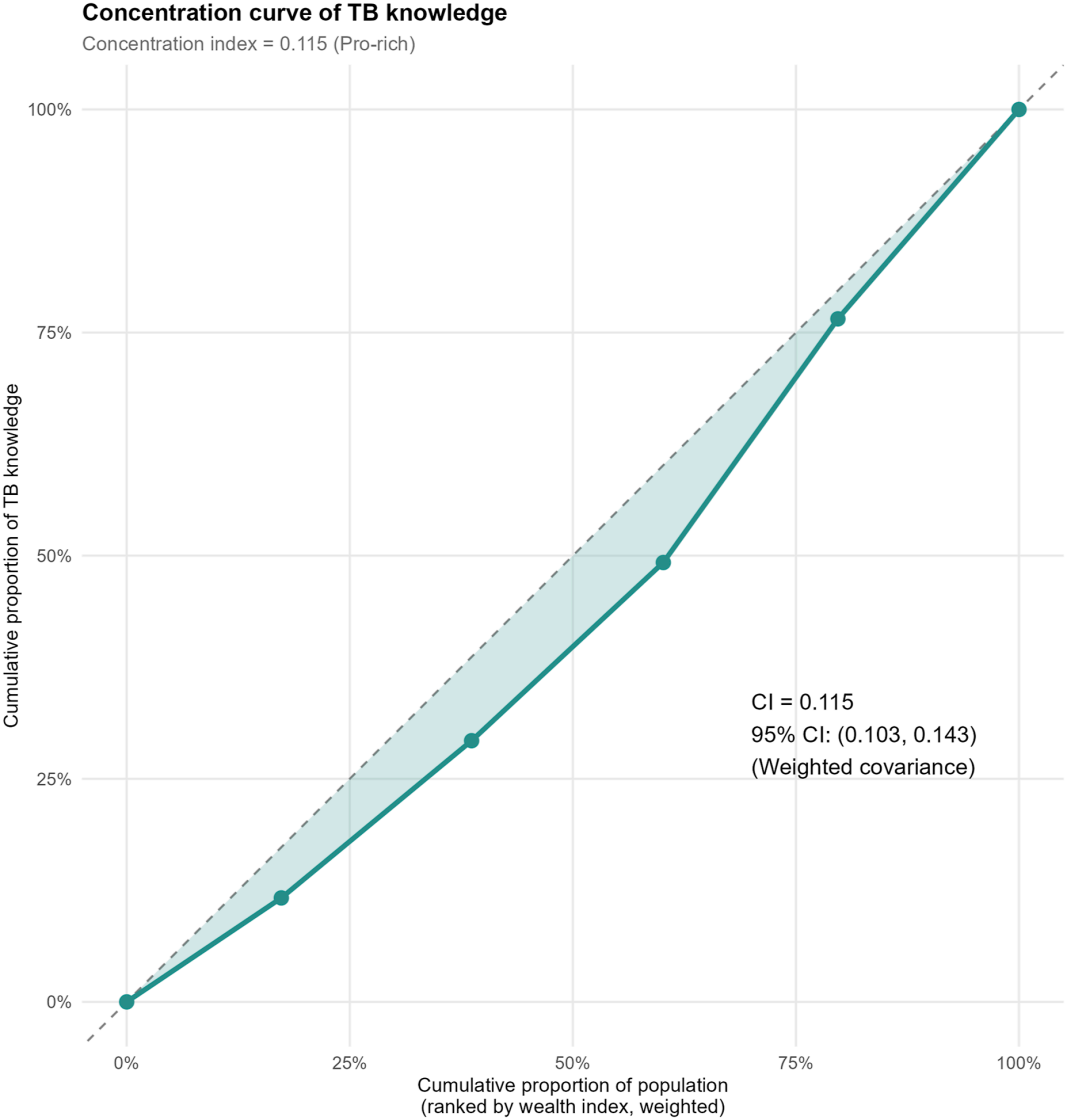
Concentration curve of comprehensive TB knowledge among women of reproductive age by household wealth index, Peru Encuesta Demográfica y de Salud Familiar (ENDES) 2024. The x-axis represents the cumulative proportion of the population ranked by wealth index (poorest to richest, survey-weighted); the y-axis represents the cumulative proportion of comprehensive TB knowledge. The dashed diagonal line represents perfect equality (concentration index = 0). The curve lying below the line of equality indicates pro-rich concentration (concentration index = 0.115; 95% CI: 0.103–0.143), meaning that TB knowledge is disproportionately concentrated among wealthier women. The shaded area between the curve and the line of equality is proportional to the magnitude of inequality. The concentration index was computed using weighted covariance with survey-weighted fractional ranks and cluster-based bootstrap CIs (1,000 replications). CI = confidence interval.

The Concentration Index of 0.115 (95% CI: 0.103–0.143, Figure 3) confirms significant pro-rich inequality in TB knowledge, exposing what may be termed a ‘double inequity’: while TB burden concentrates among economically disadvantaged populations (global concentration indices ranging from −0.10 to −0.36 for incidence),^25^ knowledge paradoxically concentrates among wealthier individuals. Similar patterns have been documented in Vietnam.^26^ In Peru, this inequity is particularly consequential given that women in the poorest quintiles face both higher exposure risk and reduced capacity to recognise and respond to TB symptoms.^4,25^ The wealth gradient, with the richest quintile showing 37% higher odds (OR 1.37, 95% CI: 1.18–1.58), further confirms that social determinants shape TB knowledge acquisition.^27^ Behavioural research in comparable settings suggests that individuals facing potential financial consequences of a TB diagnosis may engage in information avoidance, whereby people avoid threatening health information when perceived costs of knowing outweigh the benefits of acting.^28^ This hypothesis requires prospective validation; nonetheless, it points to the need to address economic determinants alongside educational interventions.

Spatial analysis revealed significant geographic clustering (Moran’s I = 0.077, *P* < 0.001), consistent with studies documenting spatial heterogeneity in TB-related outcomes.^29,30^ LISA identified 11 hotspots of high TB knowledge but no coldspots after FDR correction, suggesting that low knowledge is spatially dispersed rather than geographically concentrated. The seven low–high spatial outliers, representing clusters of lower knowledge within high-knowledge neighbourhoods, may warrant targeted attention. These findings, aligned with neighbourhood-level analyses in Lima,^30^ suggest that geographic prioritisation alone is insufficient; individual-level determinants drive knowledge gaps across all regions.

Strikingly, 41.4% of women in our sample exhibited stigmatising attitudes, a persistent barrier to care-seeking comparable to that reported in Ghana (33%)^8^ and higher than in Ethiopia (18%–24%).^31^ In addition, 54.1% perceived economic barriers to accessing health care despite free TB treatment, corroborating evidence that 43%–55% of TB-affected households experience catastrophic costs primarily driven by indirect expenses such as transportation and lost income.^32,33^ For women, these barriers are compounded by gender-specific constraints including limited financial autonomy and domestic responsibilities.^11,34^ The combined influence of stigma, indirect costs, and gendered caregiving roles may contribute to diagnostic delays and continued transmission.

Several limitations warrant consideration. Cross-sectional design precludes causal inference. Social desirability bias may have affected stigma responses. Department-level TB incidence data from 2021 were linked to 2024 survey responses, assuming relative stability in regional burden patterns. Our composite outcome definition represents one of multiple possible operationalisations, though sensitivity analyses using strict and relaxed definitions demonstrated consistent associations. Generalisability is limited to women aged 15–49 years.

## CONCLUSIONS

These findings have implications for Peru’s National TB Control Plan 2024–2026 and the WHO End TB Strategy.^3,7^ Educational interventions should prioritise women with limited formal schooling, targeting the approximately 13% without secondary education who demonstrate substantially lower knowledge. Communication strategies must address perceived economic barriers through explicit messaging about treatment gratuity, while implementing solutions for indirect costs such as transportation subsidies, as evaluated in Peru’s CRESIPT study.^22,35^ Spatial hotspots identified through LISA analysis could inform the deployment of community health workers and peer educators delivering linguistically and culturally adapted health promotion.^6^ Given the absence of spatial coldspots, interventions should address individual-level determinants across all regions. Gender-responsive programming must address stigma concerns amplified by social norms around female health disclosure.^11,34^ Integrating TB knowledge indicators into routine ENDES monitoring would enable systematic tracking of progress towards End TB.
